# Co-Occurrence of *mcr-1* and Carbapenem Resistance in Avian Pathogenic *E. coli* Serogroup O78 ST95 from Colibacillosis-Infected Broiler Chickens

**DOI:** 10.3390/antibiotics12050812

**Published:** 2023-04-26

**Authors:** Muhammad Usman, Muhammad Hidayat Rasool, Mohsin Khurshid, Bilal Aslam, Zulqarnain Baloch

**Affiliations:** 1 Institute of Microbiology, Government College University Faisalabad, Faisalabad 38000, Pakistan; 2 Faculty of Life Science and Technology, Kunming University of Science and Technology, Kunming 650093, China

**Keywords:** broilers, *mcr-1*, carbapenem, co-occurrence, avian pathogenic *Escherichia coli*

## Abstract

Avian pathogenic *Escherichia coli* (APEC) is responsible for significant economic losses in the poultry industry. This study aimed to molecularly detect carbapenem-resistant co-harboring mcr-1 avian pathogenic *E. coli* in broiler chickens infected with colibacillosis. A total of 750 samples were collected from colibacillosis-infected broilers, and conventional microbiological techniques were used to isolate and identify APEC. MALDI-TOF and virulence-associated genes (VAGs) were used for further identification. Phenotypic carbapenem resistance profiling was followed by molecular detection of carbapenem resistance genes (CRGs) and other resistance genes through PCR using specific primers. Isolates were also subjected to PCR for O typing, followed by allele-specific PCR to detect sequence type (ST) 95. Results showed that 154 (37%) isolates were confirmed as APEC, with 13 (8.4%) isolates found to be carbapenem-resistant (CR)-APEC. Among CR-APEC isolates, 5 (38%) were observed to co-harbor mcr-1. All CR-APEC showed the presence of five markers (ompT, hylF, iutA, iroN, and iss) APEC VAGs, and 89% of CR-APEC isolates displayed O78 type. Furthermore, 7 (54%) CR-APEC isolates were observed with ST95, all displaying O78 type. These results suggest that the improper use of antibiotics in poultry production systems is contributing to the emergence of pathogens such as CR-APEC co-harboring the mcr-1 gene.

## 1. Introduction

Antibiotic resistance is a global health concern, and the use of antibiotics in food animals is a leading contributing factor to this problem. Due to the high price of mutton, chicken meat is a popular alternative source of animal protein. However, the inappropriate use of antibiotics to treat infectious diseases and promote growth in poultry flocks is a common practice in developing countries, particularly in Pakistan. As a result, the misuse of antibiotics creates selection pressure and leads to the emergence of resistant poultry pathogens. These pathogens not only cause significant production and economic losses but are also associated with public health diseases [1].

Antibiotics are commonly used in livestock to maintain animal health, promote growth, and increase production. However, reports from Pakistan indicate a very high consumption of antibiotics in the broiler production system. Moreover, it has been projected that global antibiotic consumption will rise by 67% by 2030 [2]. Concerningly, most of the antibiotics used in broiler production systems in Pakistan are classified as critically important antimicrobials (CIA) by the World Health Organization (WHO) [3]. This excessive use of antibiotics hinders the control of diseases and accelerates the emergence of resistant bacteria that cause severe infections in poultry, such as colibacillosis.

Avian pathogenic *Escherichia coli* (APEC) is the causative agent of avian colibacillosis, which is associated with high morbidity and mortality rates, resulting in significant economic losses for the poultry industry worldwide. APEC’s virulence is attributed to various combinations of virulence-associated genes (VAGs) that encode for adhesins, iron scavenging, serum resistance, toxins, and more [4]. Five virulence genes, including ompT, hylF, iutA, ironN, and iss, are located on the ColV plasmid in APEC. APEC shares VAGs-relatedness with the extra-intestinal, pathogenic *E. coli*, which can infect humans [5]. Therefore, APEC could pose a potential public health concern, as well as a zoonotic risk [6]. Moreover, food-borne uropathogenic *E. coli* ST95 and ST131 are emerging strains among poultry, and they are listed as strains that can be transmitted among food-producing animals and humans [7].

*Escherichia coli* has a robust population structure that can be described by different sequence types (STs) based on phylogenetic subgroups. APEC primarily belongs to ST 95, which is listed in group B2 subgroup IX [8]. Multi-locus sequence typing (MLST) is typically used to delineate the subgroups of this pathogen [9]. However, MLST can be expensive and time consuming. In recent times, new and efficient molecular typing tools have been developed and validated. One such tool is allele-specific PCR, which has shown reliable efficacy in detecting STs related to various subgroups of *E. coli*. This technique has replaced various epidemiologic analyses. It appears that some of these subgroups are found preferentially in various extra-intestinal pathologies [10].

Poultry is a vital industry in Southeast Asia, with notable exports of poultry products coming from countries in the region. However, infectious diseases such as colibacillosis have a significant economic impact on this industry. While several studies have been reported from different parts of the region in the recent past [4,11,12], there is limited data on mcr-1-producing APEC in broilers infected with colibacillosis, particularly co-harboring carbapenem-resistance determinants and associated sequence type (ST). In this study, we report the first molecular detection of carbapenem-resistant, co-harboring mcr-1 APEC, along with ST and O type, among colibacillosis-infected broilers.

## 2. Results

### 2.1. CR-APEC O_78_ among Collected Samples

Overall, from collected samples, 418 (55.7%) were positive for *E. coli*, and among them, 154 (37%) were confirmed as *APEC*. The highest distribution of *APEC* was found in fecal samples, i.e., 47% followed by perihepatic and pericardial swabs at 31% and 28%, respectively (Table 1). Among *APEC,* a total of 13 (8.4%) were found to be *CR-APEC*. Likewise, various sample sources showed the same pattern in the case of *CR-APEC*, the highest (10%) distribution was observed in the case of fecal samples, while perihepatic swabs and pericardial swabs showed a 7% and 6% rate of *CR-APEC,* respectively. Subsequent to the phenotypic confirmation, VAGs and MALDI-TOF-based validation of the isolates was performed, and findings revealed that all 154 (100%) were confirmed *APEC* (Table 1).

Overall, 9 (69%) *CR-APEC* isolates revealed their O types, among them 8 (89%) isolates belong to O_78,_ which include 5 (55 %) from fecal samples, 2 (22%) from the perihepatic swab sample, and 1 (11%) from the pericardial swab sample, respectively. Only 1 (11%) *CR-APEC* isolate was detected with O_1_ serogroups among perihepatic samples. However, 4 (31%) out of 13 isolates have remained unassigned, as they were not detected positive for any of the tested O types.

### 2.2. Source-Wise Distribution of the mcr-1-harboring CR-APEC

Overall, a total of 61 (39.61%) isolates were observed that harbor the mcr-1 gene among confirmed *APEC*s, while 5 (3.2%) isolates were those who were detected as *CR-APEC* co-harbored mcr-1. Sample-wise distribution of mcr-1-harboring *CR-APEC* was 7% in perihepatic swabs, followed by 3% in pericardial swabs, and 1% in fecal samples (Table 2). Moreover, O serotyping results revealed that 4 (80%) mcr-1-harboring CR-ACEP belonged to O_78_ and only 1 (20%) isolate was observed with O_1_ type, while O_2_ was not detected in any of the isolates (Table 2).

### 2.3. Detection of VAGs in APEC Isolates

**Figure 1:**
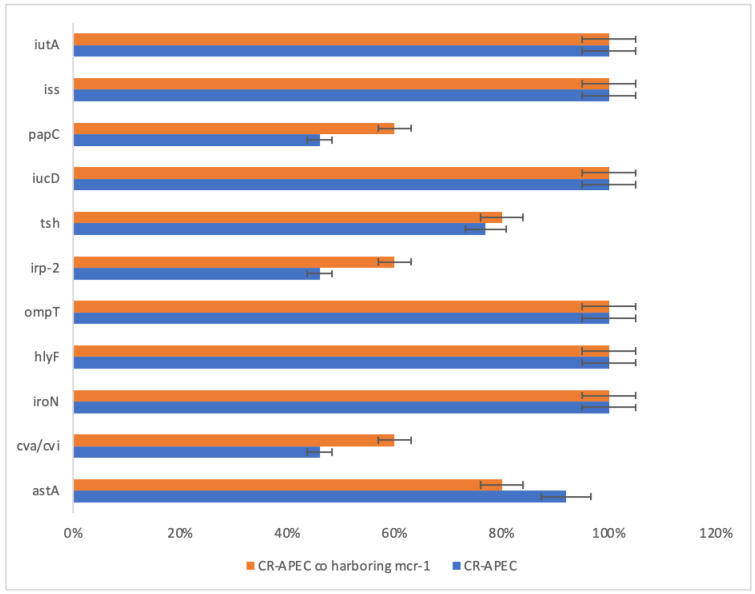
The detection rate of VAGs in CR-APEC and CR-APEC co-harboring *mcr-1* isolates.

The VAGs for *APEC* were detected in all (100%) isolates, i.e., a minimum of three VAGs were present in each of the isolates. Overall, the 28 (18%) isolates showed the presence of all studied VAGs, and 43 (28%) isolates were considered positive for eight VAGs. In addition to that, five VAGs were detected in 56 (36%) isolates, followed by four VAGs in 24 (16%) and three VAGs in 3 (2%) *APEC* isolates, respectively. In the case of five marker genes, i.e., *ompT, hylF, iutA, ironN,* and *iss*, these all were detected in 127 (82%) *APEC* isolates. Moreover, all *CR-APEC* (*n =* 13) showed the presence of five marker *APEC* VAGs. In addition to the marker genes, *iucD* was the most common VAG among *CR-APEC,* followed by *astA* and *tsh,* whereas *papC* and *cvi* were observed in 6 (46%) *CR-APEC* isolates (Figure 1).

### 2.4. Resistance Profile of the Isolates

Resistance profiling of *APEC* revealed that all 154 (100%) *APEC* isolates were found to be resistant to ampicillin and cefepime, followed by 137 (89%) to chloramphenicol, 125 (81%) to tetracycline, 101 (65%) to trimethoprim, 84 (54%) to ciprofloxacin, and 72 (46%) to colistin, respectively. Additionally, phenotypic resistance profiling of the isolates through CNPt showed that a total of 18 (11%) *APEC* isolates were confirmed as *CR-APEC* and found to be resistant to meropenem, whereas only 4 (2.5%) *APEC* isolates were found resistant to tigecycline, and among them 2 isolates were mcr-1-harboring *CR-APEC* (Table 3).

### 2.5. Distribution of ARGs in APEC Isolates

Overall, *APEC* isolates were screened for various ARGs. Findings of the molecular detection revealed that the *bla*_CTX-M_ was detected in 151 (98%) of the isolates, followed by the *bla*_TEM_ in 114 (74%) isolates, the *bla*_SHV_ in 88 (57%) isolates, the *tetA* & *tetB* in 83 (54%) isolates, the *qnrA* in 76 (49%) isolates, *qnrB* 74 (48%) isolates, and mcr-1 in 61 (39%) isolates, respectively.

**Figure 2:**
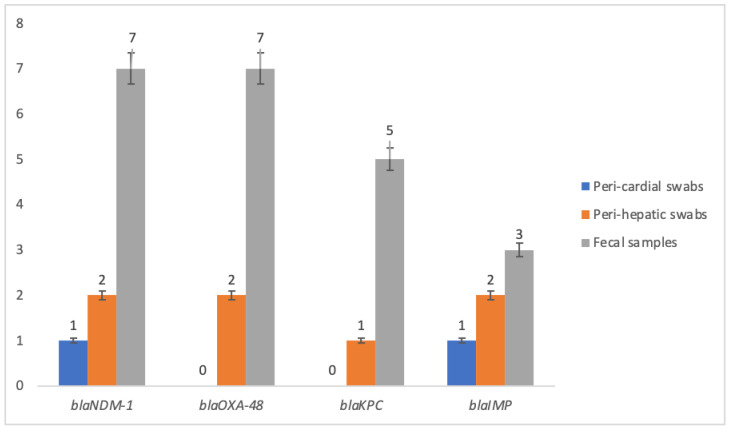
Sample-based detection of carbapenem-resistant genes (CRGs) among CR-APEC isolates.

Moreover, the highest detected resistance determinant among the *CR-APEC* isolates was *bla*_NMD-1_, which was detected in 10 (77%) isolates, which included 1 (50%) from pericardial swabs, 2 (66%) from perihepatic swabs, and 7 (87%) from fecal samples (Table 4). Conversely, the *bla*_oxa-48_ was detected in 9 (69%) isolates, which included 2 (66%) from perihepatic swabs and 7 (87%) from fecal samples. Both *bla*_KPC_ and *bla*_IMP_ genes were detected in 6 (46%) *CR-APEC* isolates, respectively (Figure 2).

### 2.6. CR-APEC ST95

Allele-specific PCR findings revealed that a total of 7 (54%) *CR-APEC* isolates were observed with an ST95 allelic profile. Among ST95, a total of 5 (71%) isolates were from fecal samples and 2 (28%) isolates were from perihepatic swabs, whereas no ST95 isolate was detected from pericardial swabs. Conversely, from mcr-1-harboring *CR-APEC*, a total of 3 (60%) isolates showed the allelic profile of ST95, among them 2 (66%) isolates were from perihepatic swabs and 1 (33%) isolate was from fecal samples. Furthermore, all 7 (100%) *CR-APEC* ST95 isolates were observed with serogroup O_78._

## 3. Discussion

Avian colibacillosis is a leading cause of substantial economic impact on the poultry industry, especially in developing countries with average production facilities. The public health significance of APEC is now recognized and has been reported from various regions of the globe [13]. Literature-based analysis has shown that colibacillosis is an important and leading disease of poultry. The aberrant use of antibiotics to treat such infections in poultry production systems has a direct influence on the emergence and spread of resistant superbugs, such as CR-APEC. Consequently, it becomes a dire health challenge that affects the well-being, health, and economic growth of developing countries [14]. Therefore, studies that delineate the virulence and resistance determinants of APEC may help to curb the damaging economic and health impacts created by this pathogen.

The current study demonstrated the molecular detection of CR-APEC co-harboring mcr-1 along with its prevalent ST (ST95) and serogroup (O78). Overall, 37% of APEC were detected from all the collected samples from various sources, and isolates were confirmed based on VAGs. These results are comparable with already published findings from Pakistan and neighboring regions, such as Bangladesh, India, and Nepal [12,15]. However, the high rate of detection in this study may be due to the use of highly sensitive tools, including MALDI-TOF and PCR-based detection of VAGs, as well as allele-specific PCR and PCR for O typing. Furthermore, differences in geographic location, sanitary facilities, sanitation systems, and additional practices in poultry production systems may be the reason for the observed variations in the findings. It is important to note that only APEC was isolated in the present study, and other pathogenic forms of *E. coli*, such as uropathogenic *E. coli* (UPEC) or sepsis-associated *E. coli* (SEPEC), may also be involved in the infection [15].

Carbapenem-resistant Enterobacteriaceae (CRE) are well-documented in various parts of the world, including Asia [16]. The present study validated that 8% of confirmed avian pathogenic *E. coli* (APEC) were carbapenem-resistant (CR-APEC), while their detection rate was about 2% among all 750 collected samples (Table 1). In addition to different antibiotic resistance genes (ARGs), various carbapenem resistance genes (CRGs) were identified in the CR-APEC isolates, including blaNMD-1 (77%), blaoxa-48 (69%), blaKPC, and blaIMP (46%) (Figure 2). Similar findings have been reported in a recent systematic review that showed the prevalence of different CRGs among livestock, such as blaNMD-1 and its variants, blaoxa-48, blaKPC and its variants, and blaIMP. A study on different avian species also showed the presence of CRE and various CRGs, such as blaNMD-1 and blaoxa-48, along with their whole genome-based phylogroup and STs [16]. Overall, various ARGs were detected in the present investigation, and a significant correlation was observed between antibiotic resistance profiling of the isolates and distribution of ARGs. For instance, the maximum resistance among APEC was recorded for Ampicillin, and blaTEM was detected in 74% of the isolates. Similarly, 46% of the isolates were found to be resistant to colistin, and mcr-1 was detected in 39% of the resistant isolates. These results are consistent with those of Liao et al., 2019, who also found a high resistance rate and different ARGs among CRE isolated from birds [16].

The imprudent use of antibiotics as growth promoters is a traditional malpractice in poultry production systems, particularly in developing countries located in Southeast Asia [3]. This practice contributes to the emergence of antibiotic-resistant bacterial strains, such as mcr-1-harboring avian pathogenic *E. coli* (APEC). The present study supported this claim, as approximately 40% of the APEC isolates showed resistance against colistin, which is considered one of the last resort antibiotics for Gram-negative bacilli (GNB). Similar findings, showing almost the same colistin resistance profiling with a 39% detection rate of mcr-1 among APEC, have also been documented from Pakistan [11]. These results are consistent with recent findings reported from Brazil, which documented a high prevalence of colistin (40%) and mcr-1-harboring APEC among commercial poultry [17]. A study from Iran also showed a much higher (68%) prevalence of colistin resistance [17]. Similarly, a study from Tunisia showed a significantly high prevalence of β-lactamase-producing APEC co-harboring mcr-1 among broilers infected with colibacillosis [18]. These findings suggest that colistin resistance among poultry is persistent, particularly in developing countries, and needs to be addressed. The inappropriate use of antibiotics as growth promoters in poultry production should be banned to prevent the emergence of antibiotic-resistant bacterial strains.

This investigation examined ten VAGs and found that all APEC isolates (100%) had at least three VAGs present. The study identified that five marker genes, including ompT, hylF, iutA, ironN, and iss, were present in 82% of APEC isolates. Moreover, all CR-APEC isolates were found to have the marker VAGs. Among CR-APEC isolates, iucD was the most common VAG, while papC and cvi were observed in 46% of the CR-APEC isolates. Previous studies from Pakistan have also reported that the iss gene was the most common VAG among APEC isolated from broilers and layers, as well as in another study conducted on APEC recovered from broilers in Pakistan. [11]. These findings are consistent with studies conducted in other regions, such as Cummins et al. (2019), who reported similar VAG profiles in a diverse APEC population from Australian commercial poultry [19]. Additionally, all the detected VAGs in this study have been previously identified as key players in APEC virulence [20].

The study investigated three types of serogroups (O1, O2, and O78), with O78 being the most prevalent (89%) and only one isolate (11%) exhibiting the O1 serogroup. The selection of these serogroups was based on their reported prevalence in avian pathogenic *E. coli* (APEC) and the available literature [21,22]. A previous study from Pakistan also reported the detection of O78, but unlike the present study, detected O2 at a rate of 13% [11]. The present study’s findings are consistent with those reported from parts of the Middle East, Europe, and Australia, which documented the detection of O78 serogroups [1,19].

By using allele-specific PCR, the study found that 54% of CR-APEC isolates belong to ST95. ST95 is a common ST among APEC, and many studies have reported its high prevalence among *E. coli* isolated from various avian sources [23]. The study’s results showing a high abundance of ST95 are not surprising, as this ST is often associated with poultry diseases and has a significant economic impact due to high morbidity and mortality rates in infected birds [24]. These findings are consistent with those reported by Jørgensen et al. (2019), who also detected ST95 in APEC. ST95 has significant population diversity and can overlap hosts between avians and humans [25]. However, the current study has limitations and lacks information about the population structure and comparative genomics of CR-APEC co-harboring mcr-1 isolated from infected broiler chickens. This information may be studied in future research endeavors.

In conclusion, the study provides evidence that the excessive use of antibiotics in poultry production systems is directly linked to the emergence of resistant superbugs, such as CR-APEC co-harboring mcr-1. The high prevalence of these antibiotic-resistant strains of APEC is a significant concern and should be taken seriously. It is imperative to discourage the irresponsible use of antibiotics in poultry farming to control the spread of these pathogens. This would be a beneficial step towards safeguarding public health. Based on these findings, it is recommended that appropriate measures be taken to address this issue to prevent the further spread of antibiotic-resistant APEC strains.

## 4. Materials and Methods

### 4.1. Ethical Approval and Study Settings

The current study was carried out after proper approval from the Institutional Review Board (IRB) and Ethical Review Committee (ERC) Ref No. GCUF/ERC/273, Date: 7-09-2020, Government College University Faisalabad. All the samples were collected after permission was granted on the prescribed form shared with the owners of the poultry farms. All the experiments were conducted in the bacteriology laboratory at the Institute of Microbiology, Government College University Faisalabad. The matrix-assisted Laser Deionization-TOF experiment was conducted at the National Institutes of Health, M4QP+GW7 Islamabad, Pakistan.

### 4.2. Sample Collection, Preliminary Screening, and Transportation

**Figure 3:**
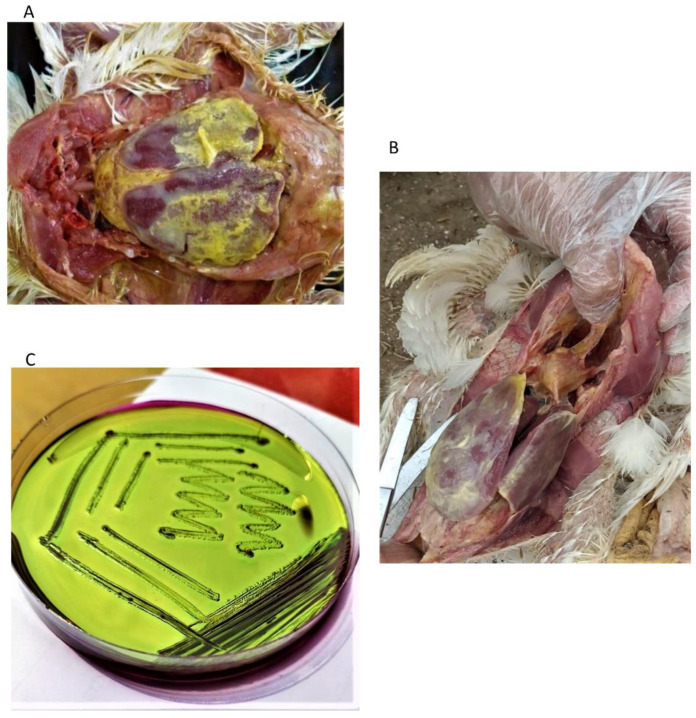
(**A**) Sampled broiler showing a typical gross lesion of colibacillosis, i.e., perihepatitis (**B**) Sampled broiler showing a typical gross lesion of colibacillosis, pericarditis, and perihepatitis (**C**) EMB agar plate showing metallic sheen colonies of *E. coli*.

A total of *n* = 750 samples were collected in sterile containers from colibacillosis-infected broilers, including pericardial swabs, perihepatic swabs, and fecal samples, 250 from each category. Samples were collected after an initial screening based on farm history, typical clinical manifestation, and colibacillosis gross lesions, e.g., perihepatitis and pericarditis observed during post-mortem examination (Figure 3A,B). Samples were enriched in 0.9% normal saline and transported at 4 °C to the bacteriology laboratory, Institute of Microbiology, Government College University Faisalabad for further investigations.

### 4.3. Isolation and Identification of E. coli

Streaking of enriched samples was conducted on MacConkey agar and EMB agar (OXOID^®^, Basingstoke, UK), and Petri plates were incubated for 24 h at 37 °C. Samples were observed for cultural (Figure 1C) and morphological characteristics. Biochemical characterization of the isolates was conducted using an API 20E kit (Biomeurex, Craponne, France) according to the manufacturer protocol.

### 4.4. Matrix-Assisted Laser Desorption/Ionization-Time of Flight (MALDI-TOF) Based Confirmation

The VITEK^®^ MS V3.2 (Biomeurex, Craponne, France) was used for MALDI-TOF confirmation of the isolates, according to the configuration and procedure guide provided by the manufacturer. The *E. coli* ATCC™8739 was briefly taken as a test control, and a total of 0.01 μL cyanohyrodxycinamic acid (CHCA) was used as a matrix for the bacterial isolate. The VITEK^®^ MS (Biomeurex, Craponne, France) slide was prepared by inoculating the control and the tested isolate using Vitek^®^ PICKME NIBS (Biomeurex, Craponne, France) in their respective and targeted circles, respectively, followed by the addition of matrix e.g., CHCA. Finally, MYLA^®^ software (Biomeurex, Craponne, France)was used to interpret the results.

### 4.5. VAGs-Based Confirmation of APEC

Overall, different VAGs were detected through PCR, using specific primers in addition to the marker VAGs for APEC, e.g., *ompT, hylF, iutA, ironN,* and *iss,* etc. The presence of a minimum of three *APEC*-specific VAGs was considered for confirmation of the isolate. For this purpose, isolates were subjected to DNA extraction using the GeneJET Genomic DNA purification kit K0722 (Thermo-Scientific™), according to the given protocol.

Subsequently, PCR (T3000, Thermo-cycler:48 Biomerta™, Göttingen, Germany) was carried out with specific conditions for VAGs, along with their respective annealing temperature given in Table 5. The reaction setup was comprised of initial denaturation of template DNA at 94 °C for 5 min, followed by 30 reaction cycles of DNA denaturation at 94 °C for 1 min, specific primer annealing for 85 s, and extension at 72 °C for 60 s. Finally, a 10 min extension was carried out at 72 °C.

The PCR reaction mixture (25 μL) contained: template DNA 5 μL, F&R primers (100 pM) 1 μL each, DreamTaq (Thermo-Scientific™, Waltham, MA, USA) 8 μL, and SuperQ water (Ambion-AM9932, Thermo-Scientific™, Waltham, MA, USA) 10 μL. For the examination of PCR amplicons, agarose (CSL-AG500; CLEAVER SCIENTIFIC^®^, Rugby, UK) gel electrophoresis was carried out and visualized under a gel trans-illuminator (BioRad, Hercules, CA, USA).

### 4.6. Antibiotic Susceptibility Testing

The Kirby–Bauer disc diffusion assay was performed to study the antibiotic resistance profile of *APEC* isolates (*n* = 154) according to the 2021 CLSI guidelines. Antibiotics used in the study include Ampicillin, cefepime, ciprofloxacin, levofloxacin, chloramphenicol, trimethoprim, imipenem, meropenem, colistin, tetracycline, and tigecycline. During the assay, *E. coli* ATCC™8739 was used as quality control. The Broth microdilution method (BMD) was used to estimate the minimum inhibitory concentration (MIC) as per CLSI guidelines, while colistin and tigecycline were estimated according to the EUCAST-CLSI and FDA recommendations, respectively.

### 4.7. Carba NP Test (CNPt-CLSI)

Phenotypic confirmation of *CR-APEC* was conducted through CNPt, according to the CLSI guidelines (CLSI 2018). A total of 100 μL of Tris-HCl lysis buffer (20 mM) was briefly added in two separate Eppendorf tubes labeled 1 and 2. With the help of a sterilized platinum loop, freshly grown bacterial colonies were dispensed in each tube. Subsequently, solution A (100 μL), made up of phenol red (0.5%) and ZnSO_4_ (0.1 mm/L) with pH 7.8, was added in tube 1 while solution B (100 μL), which was prepared by adding imipenem (6 mg/mL) to solution A, was added in tube 2. Tubes were incubated for 2 h at 37°C. Color change in the tubes was observed and the yellow color in tube B was considered positive.

### 4.8. Molecular Identification of ARGs

In addition to phenotypic-resistance profiling, various ARGs (Table 5) were identified with the help of PCR using specific primers. The DNA was extracted using a Genomic DNA purification kit K0722 (Thermo-Scientific™, Waltham, MA, USA). A total of 25 μL reaction mixture for PCR was comprised of 5 μL DNA template, 10 μL Green DreamTaq mix (Thermo Fisher Scientific, Waltham, MA, USA), and F&R primers (100 pM) 1 μL each. A total of 8 μL SuperQ nuclease-free water was added to get the desired volume (25 μL). Lastly, 1.5% agarose (CSL-AG500; CLEAVER SCIENTIFIC^®^, Rugby, UK) gel electrophoresis was carried out to examine the PCR products.

### 4.9. Serogrouping of APEC

Only *CR-APEC* (*n* = 13) were subjected to O-antigen-based serogrouping. Isolates were examined for three different and prevalent serogroups, as described previously [21], which include O1, O2, and O78. Extracted DNA was subjected to PCR using specific primers (Table 5), as described previously [21], for each of the serogroups, with thermocycler conditions as follows: a total of 25 cycles of denaturation at 94 °C for 40 s, annealing at 59 °C for 35 s, and extension at 72 °C for 60 sec. Subsequently, agarose (CSL-AG500; CLEAVER SCIENTIFIC^®^, Rugby, UK) gel electrophoresis was performed and imagined under a gel transilluminator (BioRad, Hercules, CA, USA). Additionally, gene diversity was observed with respect to the O antigen and VAGs present in *CR-APEC*.

### 4.10. Allele-Specific PCR

For the identification of ST, *CR-APEC* (*n* = 13) isolates were subjected to allele-specific PCR as developed and validated previously [10]. The DNA extraction from the isolates was carried out using a Genomic DNA purification kit K0722 (Thermo-Scientific™, Waltham, MA, USA). Afterwards, DNA was quantified using NanoDrop™ (2000/2000c, Thermo-Scientific™, Waltham, MA, USA), and 250 ng/mL was used for the further PCR reaction. A total of 25 μL PCR mixture was prepared with 5 μL extracted DNA, 10 μL Green DreamTaq mix (Thermo Fisher Scientific, Waltham, MA, USA), 1 μL of F & R *aesIX* primers (50 pM; Table 5), and 8 μL SuperQ nuclease-free water. All the isolates were examined with the *chuA* gene, which served as an internal control. The PCR was conducted with conditions as follows: initial DNA denaturation for 5 min at 94 °C, followed by 30 cycles having 94 °C denaturation for 10 s, annealing at 60 °C for 30 s, and finally, extension at 72 °C for 5 min.

### 4.11. Statistical Analysis

The study data was arranged in a Microsoft Excel spreadsheet and subjected to statistical analysis. The chi-square test was used to determine the potential relationship of the isolates with different determinants, e.g., sample source and *p*-value < 0.05 was set as significant.

## Figures and Tables

**Table 1:** Overall, sample-based distribution of APEC and CR-APEC along with their respective serogroups.

Specimen Category	No. of Samples Collected	*E coli* Positive Samples	APEC % out of *E. coli*	Overall, *APEC E. coli* Isolates Confirmation		*CR-APEC*	CR-APEC Serogroups	Statistical Analysis
				*MALDI-TOF*	*VAGs*			
Pericardial swabs	250	123 (49.20%)	35/123 (28.45%)	35 (14.00%)	35 (14.00%)	2/35 (6%)	O_78_	** *p* value = 0.001
Perihepatic swabs	250	131 (52.40%)	41/131 (31.30%)	41 (16.40%)	41 (16.40%)	3/41 (7%)	O_78_ (2) & O_1_ (1)	
Fecal	250	164 (65.60%)	78/164 (47.56%)	78 (47.56%)	78 (47.56%)	8/78 (10%)	O_78_	
Overall	750	418/750 (55.73%)	154/418 (36.84%)	154	154	13/154 (8.4%)		

** Sample wise APEC out of collected samples.

**Table 2:** Distribution of *mcr-1*-harboring CR-APEC among various sample sources along with serogroups.

Sample Source	APEC	*mcr-1*-Harboring APEC	CR-APEC co-Harboring *mcr-1*	Serogroups	ST95	Statistical Analysis
Pericardial Swabs	35	21 (60%)	1 (3%)	O_78_	ND	* *p* value ≤ 0.05
Perihepatic Swabs	41	23 (56%)	3 (7%)	O_78_ (2) & O_1_ (1)	Detected	
Fecal samples	78	17 (22%)	1 (1%)	O_78_	Detected	
**Total**	**154**	**61 (39.61%)**	**5 (3.2%)**			

* Sample-wise CR-APEC co-harboring *mcr-1 among APEC.* ND = not detected.

**Table 3:** MICs-CLSI Breakpoint based resistance profiling of *mcr-1*-harboring CR-APEC along with overall resistance profiling of APEC.

Antibiotics	Conc.	CLSI- EUCAST/FDA Resistance Breakpoints	*mcr-1*-Harboring APEC Isolates					Overall Resistance Profile of APEC
			Pericardial Swabs (*n =* 1)	Perihepatic Swabs (*n =* 3)			Fecal Samples (*n =* 1)	
Ampicillin	10 µg	≥32	256	256	256	512	256	100%
Cefepime	30 µg	≥16	128	512	256	256	128	100%
Ciprofloxacin	5 µg	≥4	64	32	64	128	32	54%
Levofloxacin	5 µg	≥4	32	128	64	128	64	54
Chloramphenicol	30 µg	≥32	128	256	128	128	128	89%
Trimethoprim	5 µg	≥16	64	256	32	64	128	65%
Imipenem	10 µg	≥4	32	128	64	64	32	11%
Meropenem	10 µg	≥4	32	64	128	64	32	11%
Colistin	10 µg	≥8	32	64	32	64	32	46%
Tetracycline	30 µg	≥16	64	128	64	128	64	81%
Tigecycline	15 µg	≥8	4	4	8	8	4	4%

**Table 4:** Co-existence of *mcr-1* and CRGs in CR-APEC among different sample sources.

Sample Source	APEC Isolates	*mcr-1* Detection	CR-APEC Detection	CR-APEC Co-Harboring *mcr-1*	Co-Existence of ARGs
Pericardial swabs	35	21 (60%)	2 (6%)	1 (3%)	*mcr-1*, *bla*_TEM_, *bla*_SHV_, *bla*_NDM-1_, *bla*_IMP_
Perihepatic Swabs	41	23 (56%)	3 (5%)	3 (7%)	mcr-1, *bla*_CTX-M_, *bla*_NDM-1_, *bla*_KPC_, *bla*_OXA-48_, *bla*_IMP_
Fecal samples	78	17 (22%)	8 (11%)	1 (1%)	mcr-1, *bla*_CTX-M_, *bla*_SHV,_ *bla*_NDM-1_, *bla*_KPC_, *bla*_OXA-48_, *bla*_IMP_
**Total**	154	**61 (39%)**	**13 (8%)**	**5 (38 %)**	

**Table 5:** Details of all the primers used in the study.

Sr. no.	Antibiotics/VAGs/Serogroups	Target	Sequence	Annealing Temp	Amplicon Size	References
ARGs						
1	β-lactams	*bla*_CTX-M-1_	F: ATGTGCAGYACCAGTAARGTKATGGC R: TGGGTRAARTARGTSACCAGAAYCAGCGG	61	593	[26,27]
2		*bla*_TEM_	F: CGCCGCATACACTATTCTCAGAATGA R: ACGCTCACCGGCTCCAGATTTAT	61	445	
3		*bla*_SHV_	F: CTTTATCGGCCCTCACTCAA R: AGGTGCTCATCATGGGAAAG	61	237	
4	Tetracyclines	tetA	F: GTAATTCTGAGCACTGTCGC R: CTGCCTGGACAACATTGCTT	55	956	[28,29]
5		tetB	F: CTCAGTATTCCAAGCCTTTG R: ACTCCCCTGAGCTTGAGGGG	55	414	
6	Quinolones	qnrA	F: TCAGCAAGAGGATTTCTCA R: GGCAGCACTATTACTCCCA	51	627	
7		qnrB	F: CGACCTGAGCGGCACTGAAT R: TGAGCAACGATGCCTGGTAG	52	515	
8	Colistin	mcr-1	F: AGTCCGTTTGTTCTTGTGGC R: AGATCCTTGGTCTCGGCTTG	60	320	[30]
9	Carbapenems	*bla*_NDM-1_	F: TGCCCAATATTATGCACCCGG R: CGAAACCCGGCATGTCGAGA	59	292	[31,32,33]
10		*bla*_OXA-48_	F: TTGGTGGCATCGATTATCGG R: GAGCACTTCTTTTGTGATGGC	55	743	
11		*bla*_KPC_	F: TGCAGAGCCCAGTGTCAGTTT R: CGCTCTATCGGCGATACCA	52	880	
12		*bla*_IMP_	F: GGAATAGAGTGGCTTAATTCTC R: CCAAACCACTACGTTATC	54	624	
**VAGs**						
1	Enteroaggregative toxin	*astA*	F: TGCCATCAACACAGTATATCC R: TCAGGTCGCGAGTGACGGC	59	116	[34]
2	Colisin V operon gene	*cva/cvi*	F: TGGTAGAATGTGCCAGAGCAAG R: GAGCTGTTTGTAGCGAAGCC	59	1181	
3	Salmochelin	*iroN*	F: AATCCGGCAAAGAGACGAACCGCCT R: GTTCGGGCAACCCCTGCTTTGACTTT	58	553	
4	Hemolysin F	*hlyF*	F: GGCCACAGTCGTTTAGGGTGCTTACC R: GGCGGTTTAGGCATTCCGATACTCAG	58	450	
5	Outer membrane protein	*ompT*	F: TCATCCCGGAAGCCTCCCTCACTACTAT R: TAGCGTTTGCTGCACTGGCTTCTGATAC	59	496	
6	Iron repressible protein	*irp-2*	F: AAGGATTCGCTGTTACCGGAC R: AACTCCTGATACAGGTGGC	58	413	
7	Temperature sensitive hemagglutinin	*tsh*	F: ACTATTCTCTGCAGGAAGTC R: CTTCCGATGTTCTGAACGT	58	824	
8	Aerobactin operon	*iucD*	F: ACAAAAAGTTCTATCGCTTCC R: CCTGATCCAGATGATGCTC	59	714	
9	P fimbriae	*papC*	F: TGATATCACGCAGTCAGTAGC R: CCGGCCATATTCACATAA	57	501	
10	Increased serum survival	*iss*	F: CAGCAACCCGAACCACTTGATG R: AGCATTGCCAGAGCGGCAGAA	57	323	
11	Aerobactin siderophores ferric receptor protein	*iutA*	F: GGCTGGACATCATGGGAACTGG R: CGTCGGGAACGGGTAGAATCG	57	302	
**O serogroups**						
1	Serogroup O1	ECO1	F: CGATGTTGAGCGCAAGGTTG R: CATTAGGTGTCTCTGGCACG	58	263	[21]
2	Serogroup O2	ECO2	F: CGATGTTGAGCGCAAGGTTG R: GATAAGGAATGCACATCGCC	58	355	
	Serogroup O78	ECO78	F: CGATGTTGAGCGCAAGGTTG R: TAGGTATTCCTGTTGCGGAG	58	623	
**ST95 Allele Specific PCR**						
1	Internal control	*chuA*	F: CGATACGGTCGATGCAAAAG R: TTGGACAACATCAGGTCATC	62	1013	[10]
2	ST95, groupIX	*aesIX*	F: CCTGGCCTGCAACGGGAG R: TCTGGCTGCGGATAAAAGAG	62	160	

## Data Availability

All the data supporting these findings is contained within the manuscript.
